# Oxytocin and bone quality in the femoral neck of rats in periestropause

**DOI:** 10.1038/s41598-020-64683-0

**Published:** 2020-05-13

**Authors:** Fernanda Fernandes, Camila Tami Stringhetta-Garcia, Melise Jacon Peres-Ueno, Fabiana Fernandes, Angela Cristina de Nicola, Robson Chacon Castoldi, Guilherme Ozaki, Mário Jefferson Quirino Louzada, Antonio Hernandes Chaves-Neto, Edilson Ervolino, Rita Cássia Menegati Dornelles

**Affiliations:** 1 Programa Multicêntrico de Pós-Graduação em Ciências Fisiológicas, SBFis, São Paulo State University (UNESP), Araçatuba São Paulo, Brazil; 2 Department of Physiotherapy, São Paulo State University (UNESP), Presidente Prudente, São Paulo, Brazil; 3 Department of Basic Sciences, School of Dentistry, São Paulo State University (UNESP), Araçatuba São Paulo, Brazil

**Keywords:** Bone quality and biomechanics, Osteoporosis

## Abstract

The objective of this study is to identify whether oxytocin (OT) contributes to the reduction of osteopenia in the femoral neck of rats in periestropause. Animals in irregular estrous cycles received two NaCl injections (0.15 mol/L) or OT (134 μg/kg) over a 12-h interval, and after thirty-five days without treatments, the biological sample collection was performed. The oxytocin group (Ot) demonstrated the highest enzymatic activity of alkaline phosphatase (p = 0.0138), lowest enzymatic activity of tartrate-resistant acid phosphatase (p = 0.0045), higher percentage of compact bone (p = 0.0359), cortical expression of runt-related transcription factor 2 (p = 0.0101), osterix (p = 0.0101), bone morphogenetic protein-2/4 (p = 0.0101) and periostin (p = 0.0455). Furthermore, the mineral-to-matrix ratio (ν_1_PO_4_/Proline) was higher and type-B carbonate substitution (CO_3_/ν_1_PO_4_) was lower (p = 0.0008 and 0.0303) in Ot group. The Ot showed higher areal bone mineral density (p = 0.0050), cortical bone area (p = 0.0416), polar moment of inertia, maximum, minimum (p = 0.0480, 0.0480, 0.0035), bone volume fraction (p = 0.0166), connectivity density (p < 0.0001), maximal load (p = 0.0003) and bone stiffness (p = 0.0145). In Ot percentage of cortical pores (p = 0.0102) and trabecular number (p = 0.0088) was lower. The results evidence action of OT in the reduction of osteopenia, suggesting that it is a promising anabolic strategy for the prevention of primary osteoporosis during the periestropause period.

## Introduction

The perimenopause period is defined by hormonal changes that impact female skeletal health and bone strength. Lower estradiol secretion provides a longer period of osteoclast activity and reduces osteoblast activity, which causes bone structure imbalances during late perimenopause and early postmenopausal years. This accelerated bone turnover rate causes changes in cortical and trabecular microarchitecture with substantial bone loss and higher incidence of fractures.

During perimenopause, around age 50, white women have a 16% risk of hip fractures and an 8% lifetime risk of death from hip fractures^1–3^. Bone microstructure analysis^4^ shows a more pronounced influence of cortical bone tissue on stiffness than the trabecular bone, which is a strong candidate for the prediction of bone strength and fractures^5^. The aging population generally exhibits a higher cortical porosity, which explains the occurrence of a fracture in the proximal femur^6,7^. Such changes on the microstructure of the bone may also related to changes of the physical and chemical properties (mineral and matrix) that occur in the tissue because severe osteoporosis is associated with decreased cortical thickness and reduced concentrations of phosphate and carbonate in this region^8,9^.

These strategies demonstrated the interference of central control in bone metabolism, such as the anabolic action of oxytocin (OT) and the existence of functional OT receptors in human osteoblasts and osteoclasts.

Innovative prevention strategies have been investigated with the aim of ensuring bone quality and quality of life. These strategies demonstrated the interference of central control in bone metabolism, such as the anabolic action of oxytocin (OT) and the existence of functional OT receptors in human osteoblasts and osteoclasts^10–13^. The action of this hormone on the skeleton is not restricted to favoring osteogenesis but extends to modulating the formation and function of osteoclasts^13^. Postmenopausal osteoporotic women have a lower OT plasma concentration^14^, possibly because of the interdependent relationship of OT and estrogen, since the steroid stimulates the synthesis of OT and its receptor, and OT acts as an anabolic mediator of the action of estrogen in the bone^15^.

Accelerated bone loss is one of the most striking occurrences in the years immediately before menopause. Therefore, it is extremely important to develop new alternatives, in perimenopause, to prevent the occurrence of osteopenia and osteoporosis. In our previous studies, we have observed that the period of alterations in the regularity of the estrous cycle in Wistar rats occurs between 17 and 18 months, with a decrease in the estrogen concentration, which characterizes the beginning of reproductive senescence in these animals, known as periestropause^16,17^. Therefore, in order to study the performance of OT as an anabolic agent and possible resource in the prevention of osteoporosis, we determined an experimental protocol to analyze the femoral neck region of females during this period^18,19^.

The aim of this study was to evaluate the peripheral action of OT in the bone remodeling process towards the prevention of bone loss in femurs of irregular cycle Wistar rats in the periestropause period.

## Results

**Figure 1:**
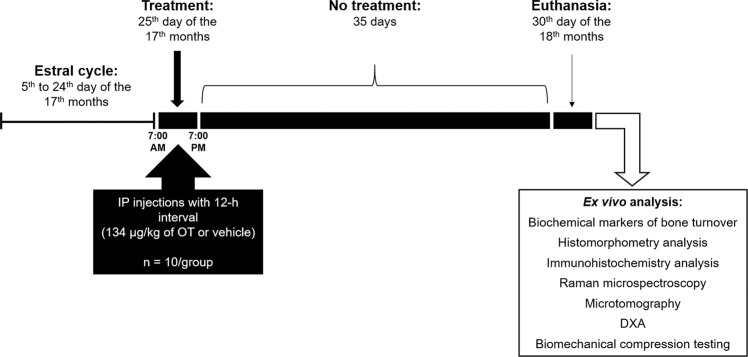
Overview of the experimental period. During the first 15 days of the 17^th^ month, the estrous cycle of the animals was analyzed. On the 25^th^ day of the 17^th^ month, the animals received a total of two intraperitoneal injections of vehicle or OT (134 µg) with a 12-hour interval (7:00 AM and 7:00 PM). After thirty-five days without any treatments (on the 30^th^ day of the 18^th^ month), the animals were euthanized, and bone and blood collection was performed.

To investigate an appropriate model of senescence, we analyzed the changes occurring in the estrous cycle of 17-month-old multiparous rats. The analyses showed that the initial change characterizing the period of periestropause in these animals was marked by increased variability in the length of the estrous cycle phases with persistent diestrus lasting 10–12 days longer with recurrence within 3 or 4 cycles. After the two OT injections, the irregularity of the estrous cycle was not significantly altered, and therefore, the experimental period was continued (Fig. 1).

### Biochemical markers of bone turnover

**Figure 2:**
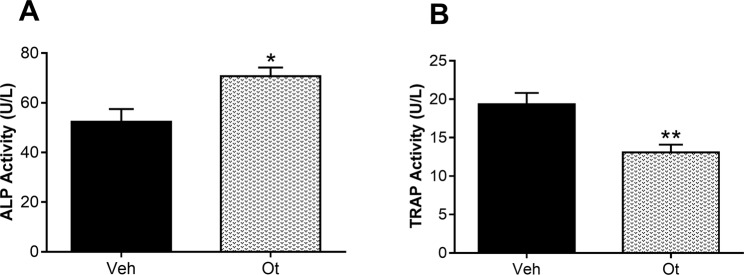
Biochemical markers of bone turnover. Activity of (**A**) alkaline phosphatase (ALP) and (**B**) tartrate-resistant acid phosphatase (TRAP) from the Wistar rats in periestropause after vehicle or OT administration. Each column represents the mean ± standard error of the mean. Statistical analysis was performed with unpaired *t*-test (*p < 0.05, **p < 0.01 vs. Veh).

After thirty-five days without any treatments, an increase in the activity of the biochemical bone formation marker alkaline phosphatase (ALP) (p = 0.0138) was verified when compared with the control group (Fig. 2A). The activity of bone resorption marker, tartrate-resistant acid phosphatase (TRAP) (p = 0.0045), was lower in rats in periestropause treated with OT in comparison to the non-treated group (Veh) (Fig. 2,B).

### Histomorphometry analysis

**Figure 3:**
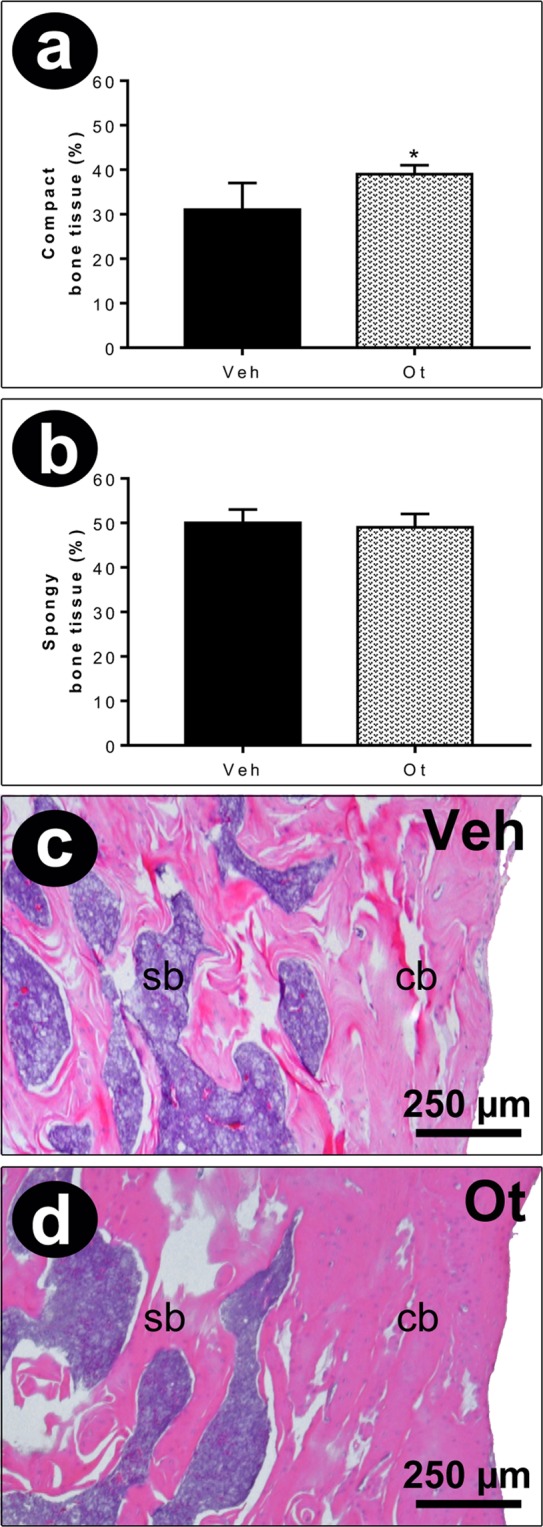
Histomorphometry analysis. Percentage of compact (**a**) and spongy bone tissue (**b**), histological sections (250 µm) of the femoral neck region (**c,d**) from the Wistar rats in periestropause after vehicle or OT administration. Each column represents the mean ± standard error of the mean. Statistical analysis was performed using the unpaired non-parametric Mann–Whitney *U* test (*p < 0.05 vs. Veh).

The percentage of compact bone tissue in the femoral neck region was 31 ± 6 and 39 ± 2 in the control and Ot groups, respectively (p = 0.0359). The percentage of cancellous bone tissue was 50 ± 3 and 49 ± 3 in the Veh and Ot groups, respectively (p = 0.8239) (Fig. 3).

### Immunohistochemistry analysis

**Figure 4:**
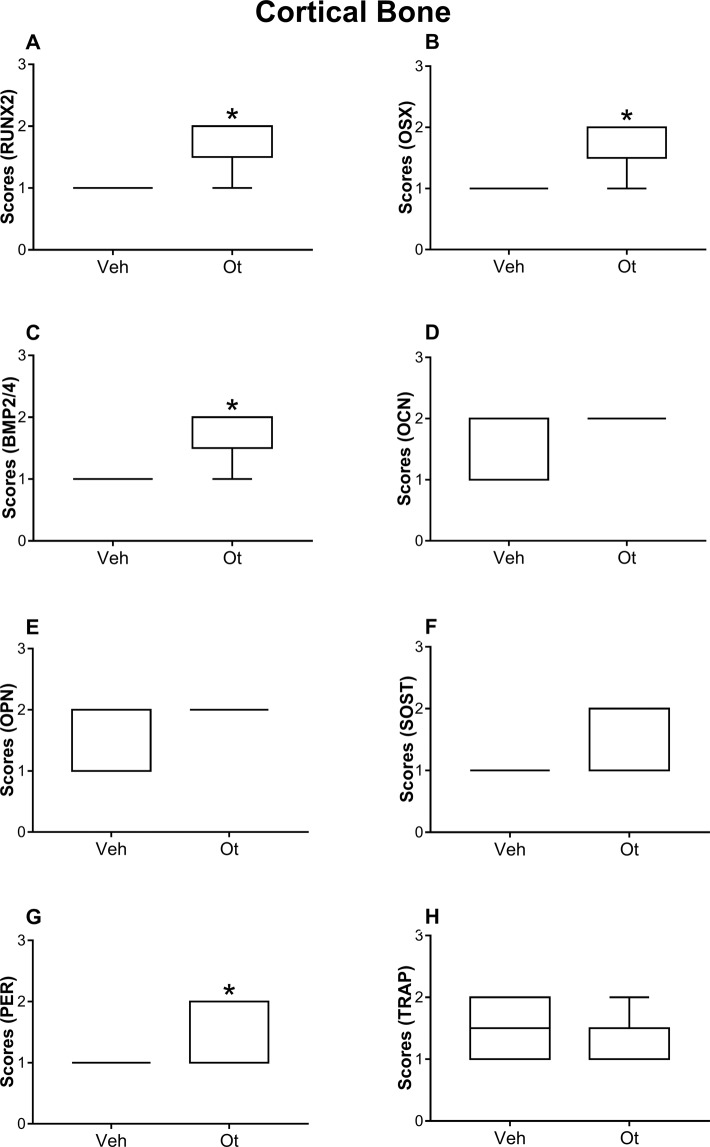
Immunohistochemistry graphs of the cortical femoral neck. Cortical bone: (**A**) RUNX2, (**B**) OSX, (**C**) BMP2/4, (**D**) OCN, (**E**) OPN, (**F**) SOST, (**G**) PER, and (**H**) TRAP from Wistar rats in periestropause after vehicle or OT administration. Each column represents the mean ± standard error of the mean. Statistical analysis was performed using the unpaired non-parametric Mann–Whitney *U* test (*p < 0.05 vs. Veh).

**Figure 5:**
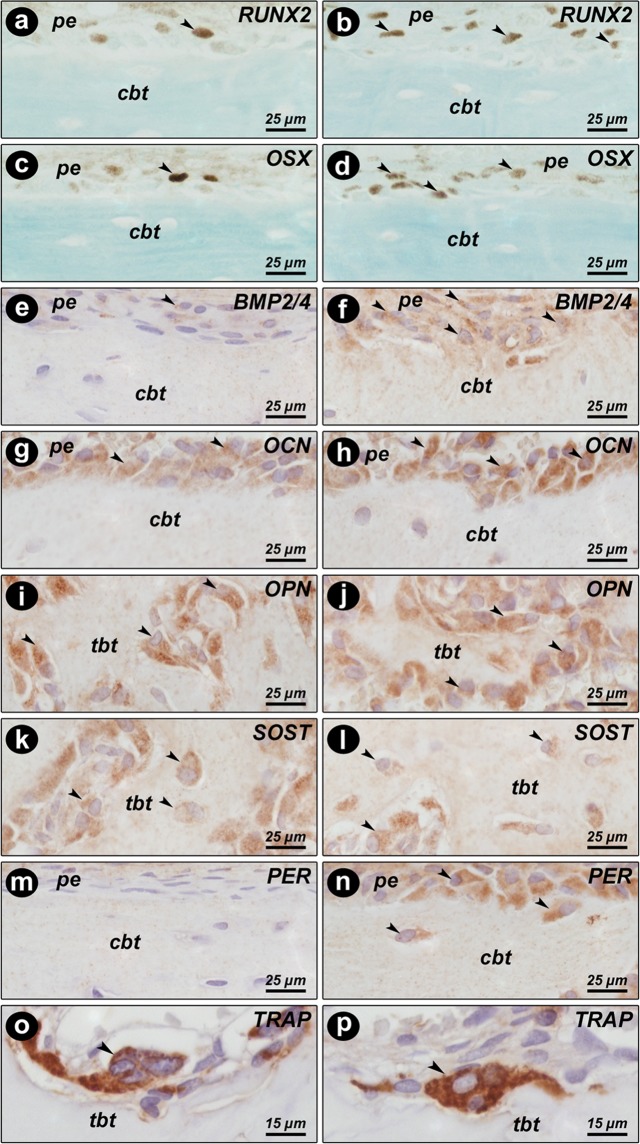
Pattern of immunolabeling for bone biomarker in the femoral neck. Photomicrographs showing the immunolabeling pattern for RUNX2 (**a,b**), OSX (**c,d**), BMP2/4 (**e,f**), OCN (**g,h**), OPN (**i,j**), SOST (**k,l**), PER (**m,n**), and TRAP (**o,p**) in the femoral neck of Wistar rats in periestropause after vehicle (**a,c,e,g,i,k,m,o**) or OT (**b,d,f,h,j,l,n,p**) administration. Abbreviations and symbols: arrows, immunolabeling cells; cbt, cortical bone tissue; pe, periosteum; tbt, trabecular bone tissue. Staining: HE. Original magnification: **a–n**, 1000×; **o,p**, 2000×. Scale bars: **a–n**, 25 μm; **o,p**, 15 μm.

**Figure 6:**
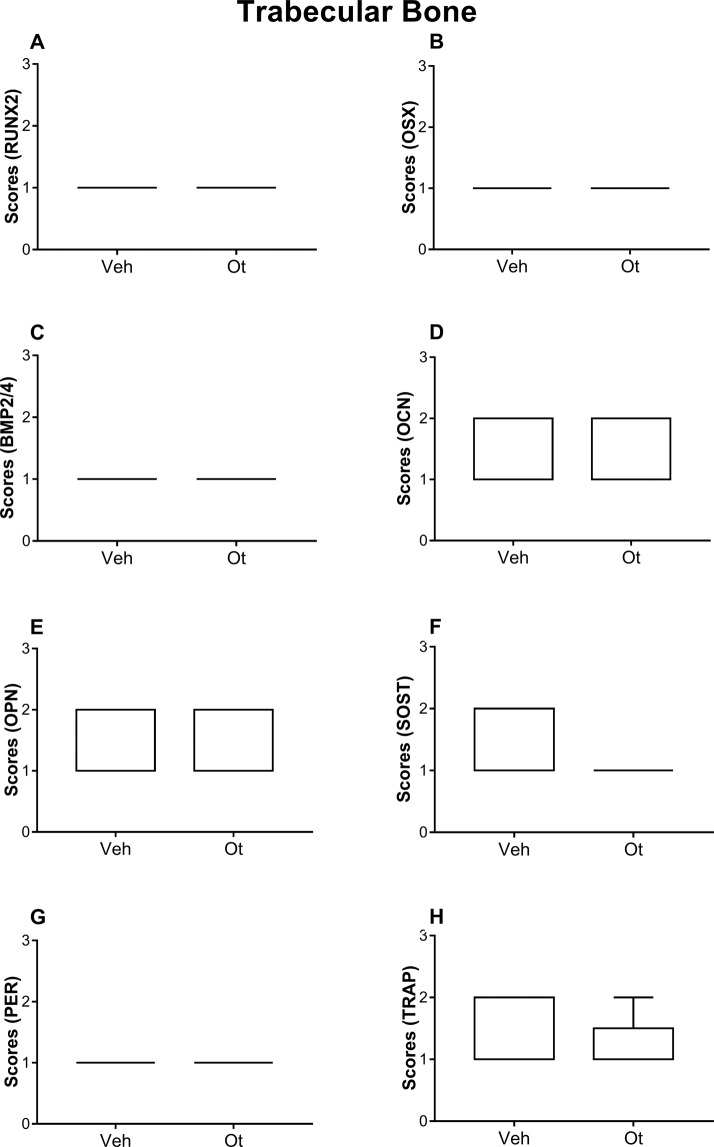
Immunohistochemistry graphs of the trabecular femoral neck. Trabecular bone: (**A**) RUNX2, (**B**) OSX, (**C**) BMP2/4, (**D**) OCN, (**E**) OPN, (**F**) SOST, (**G**) PER, and (**H**) TRAP from the Wistar rats in periestropause after vehicle or OT administration. Each column represents the mean ± standard error of the mean. Statistical analysis was performed using unpaired non-parametric Mann–Whitney *U* test.

The antibodies used in the immunohistochemical method showed high specificity for the studied proteins, which was confirmed by the complete absence of immunolabeling in the negative control. The cortical bone of the femoral neck of the animals that received OT showed a greater expression of cortical expression of runt-related transcription factor 2 (RUNX2) (p = 0.0101), osterix (OSX) (p = 0.0101), bone morphogenetic protein-2/4 (BMP2/4) (p = 0.0101), and periostin (PER) (p = 0.0455); however, in this region, osteocalcin (OCN) (p = 0.0808), osteopontin (OPN) (p = 0.0808), SOST (p = 0.1515), and TRAP (p = 0.5455) expression, was not affected by OT treatment (Fig. 4A–H and Fig. 5a–h,m,n). The same proteins in the trabecular bone did not show any significant changes (Fig. 6A–H and Fig. 5i–l,o,p).

### Raman microspectroscopy

**Figure 7:**
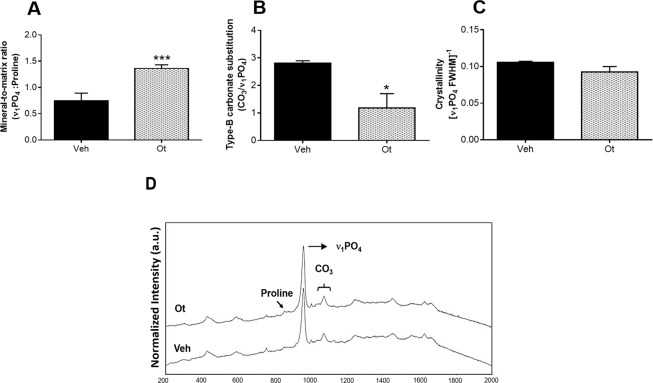
Raman microspectroscopy. (**A**) Mineral-to-collagen ratio (ν_1_PO_4_/Proline), (**B**) type B carbonate substitution (CO_3_/ ν_1_PO_4_), (**C**) crystallinity, and (**D**) representative right femur spectra from the Wistar rats in periestropause after vehicle or OT administration. Each column represents the mean ± standard error of the mean. Statistical analysis was performed using the unpaired *t*-test (*p < 0.05 vs. Veh, ***p < 0.001 vs. Veh).

The analysis performed by Raman microspectroscopy showed that the properties of the inorganic bone changed after OT treatment. The mineral-to-matrix ratio (ν_1_PO_4_/Proline) was higher and type-B carbonate substitution (CO_3_/ν_1_PO_4_) was lower in the OT group (p = 0.0008 and 0.0303, respectively) (Fig. 7A,B). Crystallinity (inverse of the full width at the half-maximum intensity of the ν_1_PO_4_ peak) did not present a statistically significant difference (p = 0.2468) (Fig. 7C). Figure 7D shows the representative right femur spectra from the Wistar rats in periestropause after Veh or OT administration.

### Microtomography

**Figure 8:**
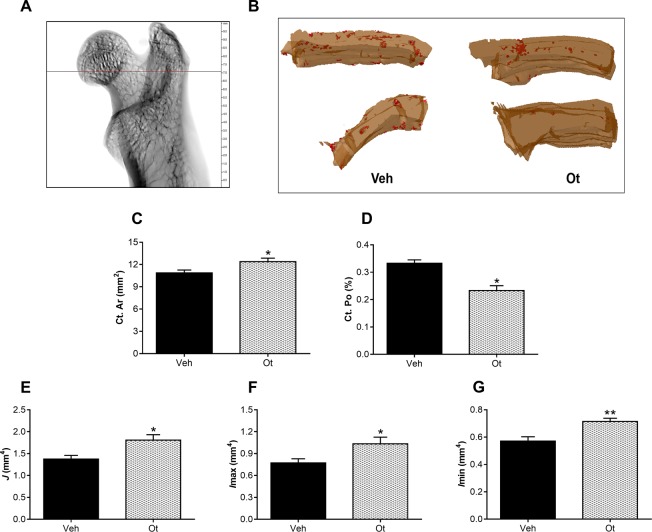
Microtomography of the cortical femoral neck. (**A**) Scanned and reconstructed image for the isolation of the femoral neck (30 slices), (**B**) 3D photomicrograph of the cortical femoral neck, (**C**) cortical bone area (Ct.Ar, mm^2^), (**D**) percentage of cortical porosity - red points - (Ct.Po, %), (**E**) maximum moment of inertia (*I*max, mm^4^), (**F**) minimum moment of inertia (*I*min; mm^4^), and (**G**) polar moment of inertia (*J*, mm^4^) from the Wistar rats in periestropause after vehicle or OT administration. Each column represents the mean ± standard error of the mean. Statistical analysis was performed with the unpaired *t*-test (*p < 0.05, **p < 0.01 vs. Veh).

**Figure 9:**
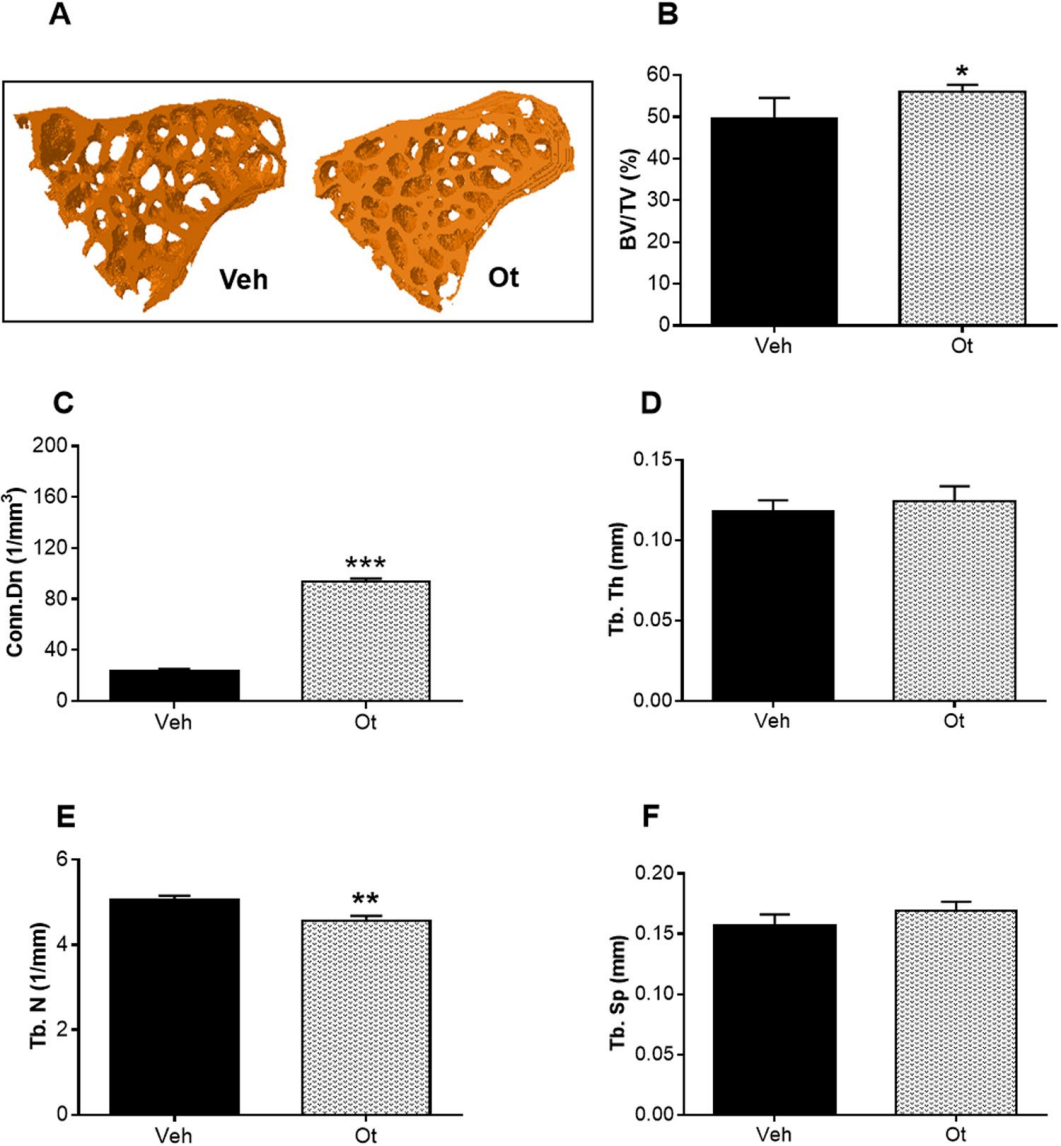
Microtomography of the trabecular femoral neck. (**A**) 3D photomicrograph of the trabecular femoral neck, (**B**) bone volume fraction (BV.TV, %), (**C**) connectivity density (Conn.Dn, 1/mm^3^), (**D**) trabecular thickness (Tb.Th, mm), (**E**) trabecular number (Tb.N, 1/mm), and (**F**) trabecular separation (Tb.Sp, mm) from the Wistar rats in periestropause after vehicle or OT administration. Each column represents the mean ± standard error of the mean. Statistical analysis was performed with the unpaired *t*-test (*p < 0.05, **p < 0.01, ***p < 0.0001 vs Veh).

The representative 3D reconstructed micro-CT images of the cortical femoral neck are shown in Fig. 8A,B. After OT treatment, changes were observed in the cortical bone. The animals exhibited an increase in the cortical bone area (Ct.Ar; mm^2^) (p = 0.0416), a decrease in the percentage of cortical porosity (Ct.Po; %) (p = 0.0102), an increase in the polar moment of inertia (*J*, mm^4^) (p = 0.0480), and maximum and minimum polar moment of inertia (*I*max and *I*min; mm^4^) (p = 0.0480 and 0.0035) (Fig. 8C–G). Figure 9A shows representative 3D reconstructed micro-CT images of the trabecular femoral neck. In the trabecular bone, the animals showed an increase in the bone volume fraction (BV/TV; %) (p = 0.0166) and connectivity density (Conn.Dn; 1/mm^3^) (p < 0.0001), and a decrease in the trabecular number (Tb.N; 1/mm) (p = 0.0088) after OT treatment (Fig. 9B,C).

### Areal bone mineral density (aBMD) and biomechanical compression testing

**Figure 10:**
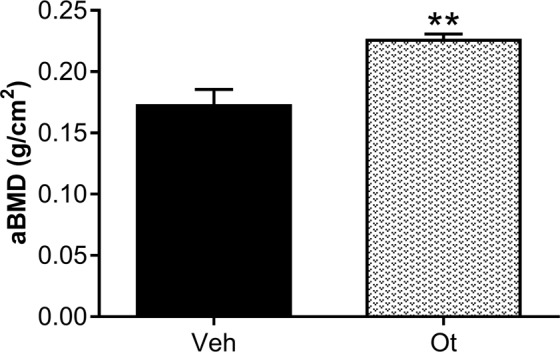
*Ex vivo* areal bone mineral density (aBMD) assessed by DXA. Areal bone mineral density (aBMD, g/cm^2^) of the Wistar rats in periestropause after vehicle or OT administration. Each column represents the mean ± standard error of the mean. Statistical analysis was performed with the unpaired *t*-test (**p < 0.01 vs. Veh).

**Figure 11:**
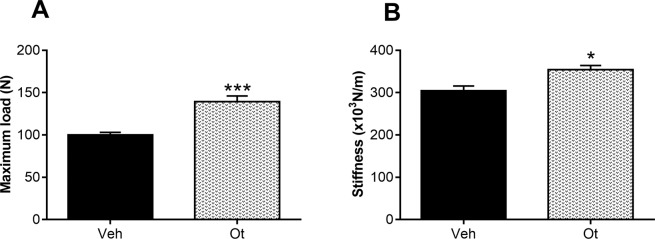
Biomechanical compression testing. (**A**) Maximum load and (**B**) stiffness of the Wistar rats in periestropause after vehicle or OT administration. Each column represents the mean ± standard error of the mean. Statistical analysis was performed with the unpaired *t*-test (*p < 0.05, ***p < 0.001 vs. Veh).

*Ex vivo* areal bone mineral density (aBMD, g/cm^2^) of the femoral neck assessed by Dual-energy X-ray absorptiometry (DXA) was found to be improved significantly after OT treatment (p = 0.0050) compared with the control group (Fig. 10). The biomechanical properties of the femoral neck are shown in Fig. 11, and they improved significantly after OT treatment. An increase in the maximum load (p = 0.0003) and stiffness (p = 0.0145) was observed as compared with the control group (Fig. 11A,B).

## Discussion

**Figure 12:**
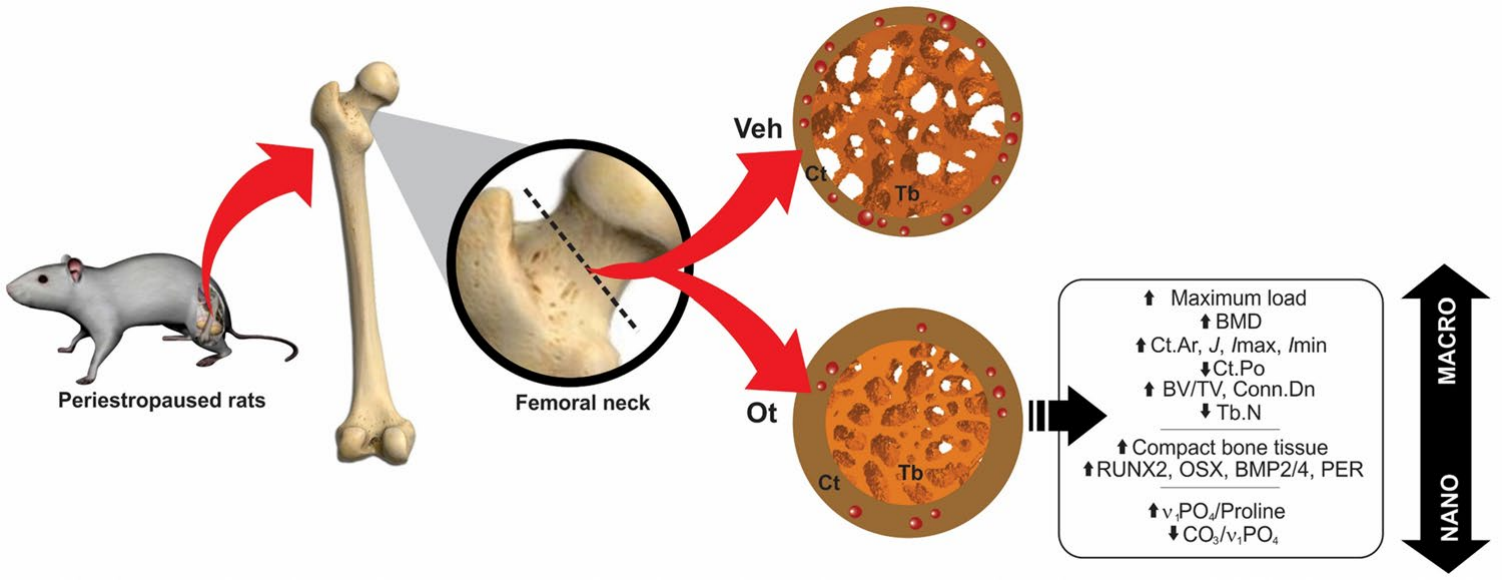
Results summary. Changes in the femoral neck of Wistar rats in periestropause after OT treatment.

Our results demonstrated that OT helps to modulate the bone remodeling cycle of senescent rats by improving the biochemical markers of bone turnover, expression of osteogenic and mineralization proteins, physicochemical properties, density, and trabecular and cortical bone microarchitecture, which culminates in a greater biomechanical response of compression (Fig. 12). The results obtained in this study are convincing scientific evidence of the action of the OT towards controlling osteopenia, suggesting a preventive effect on decreased bone mass during the periestropause period.

To investigate the action of OT in these animals, we initially analyzed the systemic bone markers. Thus, we analyzed ALP and TRAP, typical (non-specific) markers of osteoblasts and osteoclasts, which are released into the extracellular space and can be used to identify the precursors of these cells^20^. It is possible to highlight the anabolic action of OT on the bone tissue because its action on the skeleton was not only restricted to favor osteogenesis, demonstrated by the greater activity of the ALP, but also extended to a modulation of osteoclasts formation and their function, demonstrated by the lower activity of TRAP.

Data from the present study confirm our initial hypothesis that OT can act in controlling the imbalance of the activity of osteoblasts and osteoclasts mainly during the period of considerable alteration in the secretion of estradiol^16,17^, known as periestropause in rodents, similar to perimenopause in women. To understand these changes, we analyzed the transcription factors and the proteins expressed by these cells. We observed that, in addition to increasing transcription factors, such as RUNX2 and OSX, which are fundamental for osteogenesis, animals treated with OT had increased BMP2/4, a growth factor that promotes osteoblastic differentiation. A growing body of evidence suggests OT action on transcription factors and osteoinductive proteins, as evidenced by the decrease in OSX and BMP2 protein in the osteoblastic cells of young rodents OT ^− / −^^13^.

Another result of this study was the improvement in the important marker for cortical bone tissue, PER. In particular, this protein is controlled by factors such as BMP2, RUNX2, and mechanical loading, and its direct interaction with type I collagen results in bone homeostasis^21^. In addition, the positive regulation of osteoblastic characteristics occurs under the influence of cytokines and growth factors expressed with the direct or indirect orientation of BMPs acting at the transcriptional level or higher^22^. Knockout mice for PER showed a decrease in osteoblast proliferation, mineralization, attachment on the bone matrix, femoral BMD, cortical bone volume, thickness, alteration in collagen organization, and decrease in the extrinsic bone strength and intrinsic mechanical properties^23^. Furthermore, data from Kim *et al*.^24^ showed that plasmatic PER is associated with non-vertebral fractures in postmenopausal women, highlighting the importance of PER performance in the present study. The peripheral action of oxytocin promotes greater PER labeling and is associated with a greater involvement of osteoblastic cells in osteoclastic activity, which may have contributed to a better biomechanical response in these animals (higher maximum load and stiffness). These results corroborate those of another study that demonstrated that treatment with OT induced the osteoblastic differentiation of mesenchymal stem cells from the bone marrow of old female rats^25^.

The changes shown in the tissue after OT treatment through immunohistochemistry culminated in alterations of physicochemical properties (Raman microspectroscopy), and once again, there is insufficient literature on the action of OT on the physicochemical properties of bone tissue. The analysis showed that in the senescent rats treated with OT there is an increase in the mineral / matrix ratio (ν_1_PO_4_/Proline) and a decrease in the substitution of phosphate (CO_3_/ν_1_PO_4_). In this context, by decreasing the substitution of carbonate type B, animals treated with OT presented bone tissue with less maturity, however, by increasing the mineral-to-collagen ratio, these animals showed greater deposition of bone tissue, which may also explain the positive biomechanical response of bone tissue to maximum load and stiffness^26–28^.

Considering the data obtained in this study, investigating the quantity and quality of the bone tissue of these animals was essential. Bone quality results from the heterogeneous composition of bone tissue, including mineralization, morphology, microarchitecture, geometry, and microcracks, all of which are determinants of bone strength and fragility^29,30^. Taking these quantitative and qualitative findings into consideration, it is important to note that the OT action in the improvement of the trabecular region (increase in the BV/TV and the Conn.Dn) and cortical region (increase in the Ct.Ar and decrease in the Ct.Po) may have led to an improvement in the maximum load and stiffness^4,7,31^. The effects of OT verified in the data of the static histomorphometry, corroborate with the data of the cortical microarchitecture. However, the improvement in polar moments may be, mainly, related to the results of biomechanical tests. These results demonstrate that OT may favor the biomechanical properties and, therefore, decrease the occurrence of fractures. Thus, the improvement in these parameters in senescent rats that received OT is important and adds to the body of knowledge on the relevance of OT. We would like to consider the importance of future studies employing dynamic histomorphometry, mainly three-dimensional histomorphometry to demonstrate the spatial relationship between resorption cavities and formation on process of bone remodeling to obtain information on the rate of bone mineral apposition^32^.

In summary, findings such as cortical proteins, inorganic properties, BMD, trabecular and cortical quality, mechanical properties, and total ALP and TRAP in the OT group can be used as indicators that reflect the efficiency of this hormone in preventing osteoporosis. Studies on bone physiology at different stages of senescence are scarce in the literature since most studies on osteoporosis are performed in very young animals that have not yet undergone bone mass peak (9 months)^33^ and studies conducted in postmenopausal women^33^. Thus, to our knowledge, this work is a pioneering study analyzing the action of OT in the femoral neck in rats with irregular estrous cycles in periestropause.

We conclude that OT may be a promising anabolic strategy for the prevention of primary osteoporosis. It is noteworthy that the results obtained in this study are convincing scientific evidence of the action of OT in controlling osteopenia, suggesting prevention of osteoporosis in the perioperative period. Furthermore, data from this study could motivate randomized clinical and pre-clinical studies.

## Material and Methods

### Animals

Seventeen-month-old healthy female rats (n = 10/group) (*Rattus norvegicus albinus*) were housed in an environment with controlled temperature (22 ± 2 °C), humidity (55% ± 10%) and luminosity (07:00–19:00), and fed *ad libitum* (Presence Ratos e Camundongos, Paulínia, SP, Brazil). The only inclusion criteria were that the experimental animals were multiparous rats with irregular estrous cycles (periestropause) in persistent diestrus, showing at least four consecutive cycles^17^. The periestropause period in 18-month-old Wistar rats, which is similar to perimenopause in women, was described in studies by our laboratory and is characterized by a reduction in plasma concentrations of estrogen and an increase in plasma concentrations of follicle stimulating hormone (FSH)^16,17^. After confirming the irregularity of the estrous cycle, the animals were subjected to treatment with vehicle (Veh group) or OT (Ot group).

### Oxytocin and Vehicle administration

The OT was purchased from Sigma Aldrich (Sigma Aldrich, Munich, Germany), and its purity was over 97%. The OT was dissolved in 0.15 mol/L saline, and the animals received two OT doses (134 µg/Kg) administered intraperitoneally in a 12-h interval (7:00 AM – 7:00 PM) whereas the vehicle groups received a saline solution. After thirty-five days without any treatments, the animals completed 18 months and were euthanized^13,34^, according to the scheme (Fig. 1).

### Euthanasia and sample collection

Following the euthanasia, blood was collected (4 mL), centrifuged (2256 ×*g*; 15 min; 4 °C)^19^. Plasma was stored (−80 °C) to verify the cellular activity of bone metabolism. After removal, the right femurs of the experimental animals were cleaned and stored in cryogenic flasks, with a physiological saline solution, at −20 °C for microtomography (micro-CT), DXA, biomechanical compression bending, and Raman microspectroscopy. Twenty-four hours before the micro-CT, the femurs were removed from freezer and placed in the refrigerator (4 °C) to defrost; 6 hours prior to the micro-CT, they were left at room temperature. After the micro-CT process, the bones were put in the freezer again. This defrosting was performed once again for DXA, Raman spectroscopy, and biomechanical assay analysis. The left femurs were removed and processed for histomorphometry and immunohistochemical analysis. All the bones analyzed in this study went through the same defrost stages.

### Assays of biochemical markers of bone turnover

The analysis of alkaline phosphatase (ALP) and tartrate-resistant acid phosphatase (TRAP) was determined by colorimetric assay^35^. ALP was analyzed using a reaction mixture with 2.5 mmol / L *p*-nitrophenyl phosphate (*p*NPP), 2 mmol/L MgCl_2_ and 25 mmol / L glycine buffer (pH 9.4). TRAP activity was determined by colorimetric assay using a reaction mixture consisting of 10 mmol / L of *p*NPP, 50 mmol / L of sodium tartrate, 1 mmol / L of p-hydroxy-mercury benzoate (*p*HMB) and 100 mmol / L of acetate buffer sodium (pH 5.8). For both enzymes, one unit of enzyme activity was defined as the amount of enzyme required to hydrolyze 1 µmol of *pNPP* per minute at 37 °C. In these analyzes we use enzyme-free controls in each assay to adjust the non-enzymatic hydrolysis of *p*NPP ^19.36^.

### Histomorphometry analysis

Samples were processed conventionally and stained with hematoxylin and eosin^36^. In the femoral neck, the total area was observed, and the volume occupied by compact bone and spongy bone tissue was measured from this area.

### Immunohistochemistry analysis

Immunohistochemical processing was conducted using an indirect immunoperoxidase method as described by Ervolino, *et al*.^36^. The histologic slides containing samples from all experimental groups were divided into eight batches, and each batch was incubated with one of the following primary antibodies (Santa Cruz Biotechnology, TX, USA): rabbit anti-RUNX2 (SC-10758), goat anti-OSX (SC-22538) rabbit anti-BMP2/4 (SC-9003), rabbit anti-PER (SC-67233), goat anti-OCN (SC-18319), goat anti-OPN (SC-10593), goat anti-TRAP (SC-30833), and rabbit anti-SOST (ORB100911, Biorbyt, San Francisco, CA, USA). Histological sections were examined blindly under bright field illumination on a light microscope (Optiphot-2, Nikon, Japan) by investigators and a certified histologist experienced in these analyzes (E.E.). The scores were assigned twice blindly, and on different days to increase the reliability of the obtained data. Two areas were analyzed at the cortical and medullary portions of the bone tissue of the femoral neck. Each of these areas was 300 × 400 μm, and the analysis was performed at 400× magnification. The immunolabeling pattern was evaluated through scores adapted from the criteria established by Stringhetta-Garcia, *et al*.^18^. For RUNX2, OSX, and TRAP: absence of immunolabeling = SCORE 0; low immunolabeling pattern with less than three immunoreactivity cells per area = SCORE 1; moderate immunolabeling pattern, with three and seven immunoreactivity cells per area = SCORE 2; high immunolabeling pattern, with more than seven immunoreactivity cells per area = SCORE 3. Only multinucleated TRAP-positive cells near a bone surface were quantified. For BMP2/4, PER, OCN, OPN, and SOST: total absence of immunolabeling = SCORE 0; low labeling, considering that approximately 1/3 of the cells were immunoreactive and there was light labeling in the extracellular matrix: SCORE 1; moderate labeling, considering that approximately 1/2 of the cells were immunoreactive and there was moderate labeling in the extracellular matrix = SCORE 2 indicates; high labeling, considering that approximately 2/3 of the cells were immunoreactive and there was strong labeling in the extracellular matrix = SCORE 3.

### Raman microspectroscopy

The mineral and matrix composition of bone tissue was analyzed by Raman microspectroscopy using micro-Raman spectrograph (Renishaw, inVia model). The wavelength of the laser used was 633 nm at 50% of the laser power (microwatts - μW). Adjustments were made at 1800 lines per mm with an exposure time of 30 seconds, and the accumulation number was three. During the analysis process, a Leica optical microscope (DMLM series) attached to the spectrograph and the 50x objective was used. Three spectra (scans) of each sample were collected to determine the composition and relative intensities of the mineral and matrix in the neck of the right femur^37^. The Raman spectra of each sample were truncated (200 to 2000 cm^−1^), corrected at the baseline and average. The bands used in this study were phosphate (ν_1_PO_4_: 959–960 cm^−1^), collagen specific (proline: 855 cm^−1^) and carbonate (CO_3_: 1070–1077 cm^−1^)^26,27^. Based on the ratios of peak intensities the mineral-to-collagen ratio (ν_1_PO_4_/Proline) and type B carbonate substitution (CO_3_/ν_1_PO_4_) were calculated^27^. Crystallinity was determined as the inverse of the full-width at half maximum, in which the maximum was the normalized peak intensity (one) relative to the baseline (zero) of the ν_1_PO_4_ peak^8^.

### Microtomography

Bone microtomography analyzes were performed on the SkyScan 1272 microtomograph (SkyScan, Belgium) according to the protocol from Stringhetta-Garcia, *et al*.^19^

- Cortical parameters: cortical bone area (Ct Ar; mm^2^), percentage of cortical porosity (Ct.Po; %), polar moment of inertia (*J*, mm^4^), and maximum and minimum polar moment of inertia (*I*max and *I*min; mm^4^).
- Trabecular parameters: bone volume fraction (BV/TV; %), connectivity density (Conn.Dn, 1/mm^3^), trabecular thickness (Tb.Th; mm), trabecular number (Tb.N; 1/mm), and trabecular separation (Tb.Sp; mm).

The parameters were analyzed in 30 slices in the femoral neck region according to standard procedures^38^. The operator conducting the scan analysis was blinded to the treatments associated with the specimen.

### *Ex vivo* areal bone mineral density (aBMD) assessed by DXA

To evaluate whether OT could influence the bone strength, we assessed aBMD of femurs using the dual-energy X-ray absorptiometry (Lunar DPX Alpha, WI, USA), with software for measuring BMD in small animals. The equipment was calibrated according to the instructions of the manufacturer. In this way, the thawed femurs were positioned in the frontal plane and anterior-posterior view on the scanner table, oriented the same direction, and fully scanned in a bowl with 2 cm of water. A region of interest (ROI) in the neck of the femur was delimited in a square area of 0.72 mm, and all analyzes performed by the same researcher.

### Biomechanical compression testing

The evaluation of the biomechanical properties of the right femur was performed through compression tests of the femoral neck (Universal Testing Machine; DL3000, EMIC, SP, Brazil)^39,40^. The bones were placed in the metal apparatus and fixed vertically (long axis) and compression force was applied on the femoral head. This positioning allows the force generated to be parallel to the long axis of the femur, which results in bending motion in the femoral head and neck. In this way, the maximum load and stiffness could be analyzed. Biomechanical tests with a 20 mm distance between the support surface and a load application speed (strength) of 0.25 mm/min were performed. The biomechanical analysis evaluated the displacement × force curve, bone strength, the maximum load (N) accepted by the bone tissue, and stiffness (x 10^3^ N/m); the distance between the support surface was 20 mm and a load cell of 2000 N was used. The values were recorded in the manufacturer’s own computer system (Instron), which directly provides the maximum strength admitted by the femur.

### Statistical analysis

The results were analyzed using GraphPad Prism, version 6.0, expressed as the mean ± standard error of the mean (SEM). The normality of data was analyzed by the Shapiro-Wilk test; statistical comparisons were drawn using unpaired non-parametric Mann–Whitney *U* test or unpaired parametric Student’s t-test. For all analyses, *p* < 0.05 was considered statistically significant.

The groups in this study included ten rats each, and the primary variable Ct.Ar was used to calculate the power of the study using the online software OpenEpi (96%). (http://www.openepi.com/Power/PowerMean.htm).

### Institution that approved the experimental protocols

Animal procedures were approved by the Ethics Committee on Animal Use São Paulo State University (Unesp), School of Dentistry, Araçatuba (Protocol number 2014–01379) and performed in accordance with the Guide for Care and Use of Laboratory Animals.
